# Improving Laboratory-Based Cancer Drug Discovery Study Designs for Better Research Translations

**DOI:** 10.3390/mps9020038

**Published:** 2026-03-03

**Authors:** Sivananthan Manoharan

**Affiliations:** Molecular Pathology Unit, Cancer Research Centre, Institute for Medical Research, National Institutes of Health, Ministry of Health Malaysia, Setia Alam, Shah Alam 40170, Selangor, Malaysia; sivananthan@moh.gov.my

**Keywords:** basic cancer research, cancer study designs, drug discovery, preclinical cancer models, translational research

## Abstract

The process of developing a drug is complex and involves many steps, from basic research (bench) to patient applications (bedside), which are conducted to ensure the drug is both safe and effective. In cancer research, the failure rate is high when translating basic findings to clinical trials. One of the main factors probably contributing to high failure rates is the basic quality of in vitro and in vivo study designs. Advanced basic cancer research techniques, including various types of 3D cell culture, the use of valuable organoids, organs, or tumors on chips, traditional or automated Western blots, omics research, advanced imaging techniques, usage of cutting-edge preclinical models and others, may produce inaccurate results for translational research if the basic study design is not carefully planned, especially when drugs or compounds are involved. In this manuscript, the author discussed (i) the importance of understanding and applying pharmacokinetic data in basic research, (ii) a proper comparison of the efficacy and safety of investigational drugs with the standard of care, (iii) the importance of following the actual route of drug administration as experienced by patients, the cruciality of human-to-animal dose conversion, and dose frequencies in animal models, (iv) significance of the age, gender, and strain of mice, along with adherence to the ARRIVE guidelines for ensuring transparency in conducting and reporting preclinical research, (v) benefits of having both subcutaneous and metastasis models in preclinical studies, (vi) the impact of comorbidities and related cancer drug studies in animal models and (vii) the importance of testing drug candidate/s in model mimicking acidic tumor microenvironment.

## 1. Introduction

The failure rate for translating drugs from animal testing to human treatments remains over 92%, a figure that has stayed consistent for the past few decades [1]. According to the National Institutes of Health (NIH), 80 to 90% of research projects fail before reaching human trials [2]. Even if drug candidates advance to phase 1 clinical trials, 9 out of 10 will fail in phase 1, phase 2, or phase 3. The main reasons for these failures are (i) lack of drug efficacy in patients (40–50%) and (ii) high toxicity (30%) [3]. In laboratory cancer research, both in vitro and in vivo studies are essential. Before entering the preclinical testing and validation stages, scientists rigorously evaluate anti-cancer drug candidates through a series of in vitro studies. A thorough understanding of the drug candidates before starting experiments, along with well-designed and reliable in vitro study protocols, is vital for predicting the potential for successful translation. The data collected from in vitro studies provide a critical foundation for subsequent tests, such as efficacy and safety evaluations in preclinical studies, which involve valuable live animals. The design of preclinical studies must be thorough and closely simulate the conditions experienced by cancer patients in hospitals. Ensuring the robustness of both in vitro and in vivo study designs is expected to decrease the failure rate of translational research. This article highlights and discusses seven key topics aimed at addressing existing gaps and further improving both in vitro and in vivo studies. The topics are:

1. The importance of the pharmacokinetic data of established drugs/compounds,
2. Investigational compound/drug versus proven drug(s) for cancer treatment,
3. Route of drug administration, dose conversion, and frequency of drug administration in preclinical studies,
4. The age, gender, strain of mice, and the importance of the ARRIVE guideline in preclinical studies,
5. Subcutaneous versus metastatic mouse models in preclinical studies,
6. Comorbidities and related drug studies in preclinical models,
7. The acidic tumor microenvironment (TME) and the efficacy of the drug candidate.

## 2. The Importance of the Pharmacokinetic Data of Established Drugs/Compounds

Product development, especially for drugs, is undeniably one of the most complex tasks in pharmaceutical research. The traditional drug discovery process is laborious and expensive. To overcome these challenges, another strategy for drug discovery is drug repurposing, where an approved drug for one indication is redirected or restudied for a different one, such as anti-cancer activity. This approach is appealing because it reduces the overall time needed to develop an effective drug while maintaining low costs, high efficiency, and a lower risk of failure. Drug repositioning also provides additional data on the clinical aspects of the drug, such as pharmacokinetics, pharmacodynamics, and toxicity profile [4]. Besides approved drugs, many natural compounds also have similar profiles, as mentioned above. Parameters like maximum concentration (Cmax) and half-life (t_1/2_) are valuable information to consider before starting a study. We can use this critical data to design both in vitro and in vivo studies. For example, using a drug repurposing platform, pyrimethamine, an orally bioavailable old antimalarial drug, was shown to inhibit the proliferation of many types of cancer in both in vitro and in vivo studies [5,6]. The Food and Drug Administration (FDA) approved a 50 mg/day pyrimethamine dosage for humans. In a clinical trial, this dosage in leukemia patients resulted in a Cmax of only 6 µM to 10 µM. When 50 mg/day of pyrimethamine was tested in 11 subjects with HIV and Toxoplasma gondii, non-compartmental pharmacokinetic analysis showed a mean Cmax of 8 µM, while compartmental analysis indicated 6.2 µM [7]. Note that these values were calculated using a molarity and concentration calculator [8]. Based on this data, in vitro tests should not exceed 10 µM, although the IC_50_ after 3 days of cell incubation with pyrimethamine was ≥30 µM. To prevent overestimating effects on affected pathways, lower doses should be used in Western blot studies. One study employed 32 µM and 100 µM doses to examine pyrimethamine’s in vitro effects on prostate cancer cell lines [9], but testing at <10 µM would be more meaningful. The t_1/2_ of pyrimethamine is 5–6 h in mice but 96 h in humans, which likely affects efficacy and toxicity outcomes across species. The drug clears rapidly from mice but accumulates in humans with once-daily dosing, indicating that higher concentrations may be necessary in mice compared to humans. An animal dose conversion suggests about 10 mg/kg of pyrimethamine should be administered orally, consistent with FDA directions, yet many studies have used doses exceeding this, up to 75 mg/kg [6]. Doses above 30 mg/kg should be avoided, as 30 mg/kg already exceeds three times the human equivalent dose, and pyrimethamine is toxic.

Another example of repurposing is colchicine, an orally bioavailable anti-gout drug, being investigated in cancer research. Based on available data, a 1.8 mg dose of colchicine results in a Cmax of approximately 6.2 ng/mL or 15 nM, with a long half-life [10]. Note that 6.2 ng/mL was converted to 15 nM using a molarity and concentration calculator. Colchicine has a narrow therapeutic window and high toxicity risk with overdose [11]. If testing on cancer cells for 72 h yields an IC_50_ of 0.5–1 µM, researchers should avoid using this concentration for further experiments. Instead, they should use 15 nM for follow-up analyses like clonogenic, migration, and invasion assays and flow cytometry. Testing at 0.5–1 µM overestimates achievable blood concentrations and poses significant toxicity risks, which could mislead the scientific community and the public.

There are thousands of natural compounds isolated from natural resources, and many of them have pharmacokinetic, pharmacodynamic, and safety profiles. Common problems with natural compounds are their low Cmax, oral bioavailability, and short t_1/2_. When designing an experiment using parent natural compounds (without specific drug delivery systems), researchers must be aware of these issues if the compound is intended for oral administration. Only a few natural compounds have acceptable oral bioavailability and t_1/2_, making them suitable for once- or twice- or thrice-daily oral dosing. Unlike intravenous administration, oral dosing is important for the convenience and compliance of patients. Many natural compounds are reported to have in vitro anticancer effects, but many are not orally bioavailable when intended for oral use. Researchers should conduct an information search about the compounds before starting a project. If the compound has a long t_1/2_, like 20 h, it is acceptable to use the Cmax concentration to test the drug in vitro. However, if the t_1/2_ is only 4 h, researchers need to estimate the drug concentration in the blood at the 24th hour (for a single daily dose). For example, a natural compound, rocaglamide, given at 5 mg/kg orally in mice produced an 800 nM Cmax, with plasma concentrations of 20–50 nM at 24 h [12]. In this case, researchers estimating a once-daily dose in humans should focus the in vitro test on approximately 20–50 nM and avoid using 800 nM as the test concentration. Note: Although Chang et al. [12] conducted a single-dose pharmacokinetic study using rocaglamide at a dosage of 5 mg/kg orally, the same article reported that the maximum tolerated dose (MTD) for oral administration in NSG mice was 1.2 mg/kg. The disparity between 1.2 mg/kg and 5 mg/kg is significant, and achieving concentrations of 20–50 nM is likely not feasible at the 1.2 mg/kg dosage. It remains unclear why the authors did not perform pharmacokinetic studies at 1.2 mg/kg to obtain accurate values, opting instead for the 5 mg/kg dosage, which is toxic, although a single dose may not result in weakness or death within 24 h. Iwasaki et al. [13] indicated that the therapeutically significant concentration of rocaglamide is 30 nM, primarily based on in vitro studies of rocaglates of which rocaglamide is a component. For new compounds with limited information, it is advisable to determine the IC_50_ and avoid using concentrations above 10 µM. It is best to test whether the compound has activity below 10 µM, even if the IC_50_ exceeds 10 µM, since limited oral bioavailability is always a barrier to reaching the desired Cmax. Testing at low doses, like 0.5–1 µM, is recommended. Some compounds with high IC_50_ values may still produce significant cytotoxic effects when tested at doses lower than the IC_50_. This approach may work for individual compounds or in compound-compound or drug combination studies. Based on the author’s experience, this type of compound increased the cytotoxicity of cancer cells when combined with standard chemotherapy.

Another aspect to consider is the prodrug’s impact on in vitro and in vivo studies. The term “prodrugs” refers to drug derivatives that are bio-reversible, inactive, and capable of converting to their parent drug inside the body. Once considered a last resort, prodrugs are now included from the very beginning of the drug development process. Prodrugs make up about 10% of all commercially available medications worldwide [14,15]. Capecitabine, an orally administered prodrug of 5-fluorouracil (5-FU), is converted into active metabolites in the liver before being released into the gut to exhibit an anti-tumor effect. Such a prodrug could only be evaluated for effectiveness and pharmacokinetic properties using preclinical animal studies, and they were generally hard to test quickly using basic cell models in vitro unless a reliable co-culture in vitro model using hepatic cells is developed [16]. Testing capecitabine directly in vitro without a co-culture model may lead to inaccurate results that could be interpreted differently in vivo. For instance, in vitro synergism testing between compound A and capecitabine using a simple in vitro model (not a co-culture model) likely yields unreliable outcomes, and if no synergism is observed, researchers might decide not to pursue this prodrug. Such decisions could cause the loss of a potentially viable drug candidate if it is not tested in vivo. Oral administration of indole-3-carbinol (I3C) results in the conversion of the compound to diindolylmethane (DIM) and other oligomers, which are catalyzed by the acid in the stomach [17]. Based on this information, it seems that DIM is the primary active agent, whereas I3C should be considered a precursor or “pro-drug” in vivo [18]. Since the presence of stomach acid is important for converting I3C, testing the compound in an in vitro setting might be less accurate. Furthermore, conducting in silico experiments on this type of compound may result in an inaccurate shortlist of the hit compound.

In silico methodologies for drug development offer an exciting/fascinating, straightforward, and cost-effective alternative. In silico analysis methods eliminate the need for chemical compound isolation, enabling the assessment of health benefits prior to synthesis or purification of compound of interest. In silico methodologies offer tools to get an understanding of the interactions that occur between biological molecules on a systemic level via network pharmacology analysis. This method helps scientists to understand how drugs work, find new drug targets, anticipate the side effects, and find possible drug–drug interactions [19]. Computational approaches have led to the effective development of several new drugs. For example, an anticancer drug, Raltitrexed, was developed through an in silico approach [20]. Raltitrexed was shown to have binding energy of −16.57 kcal/mol to human thymidylate synthase through a molecular dynamic analysis [21]. Picomolar binding affinity is equivalent to −16.4 kcal/mol, while nanomolar affinity translates to −12.3 kcal/mol in Gibbs energy. A binding energy ranging from −12 to −16 kcal/mol is the typical target for lead optimization [22]. While in silico methods offer numerous advantages, they also come with several drawbacks. Sadybekov & Katritch [23] in Nature mentioned that “one should not forget here that any computational models, however useful or accurate, may never ensure that all of the predictions are correct.” The absorption, distribution, metabolism, excretion, and toxicity (ADMET) and pharmacokinetics profiles of compounds are crucial for effectively translating in silico findings into practical applications. For instance, if a scientist selects compound I3C for molecular docking analysis, the results may show that I3C (the ligand) interacts well with the protein of interest, such as BCL3. However, preclinical studies are unlikely to replicate similar results, despite the accuracy of these predictions if I3C is designed for oral delivery. This is because, as discussed above, the I3C will undergo extensive metabolism following oral administration, leading to the release of various metabolites, with DIM identified as one of the primary metabolites present in the blood. It has been reported that I3C is not detectable in the circulation after one hour post oral administration [17]. Many natural compounds often exhibit low or poor oral bioavailability and/or are metabolized extensively, which can negatively impact their efficacy despite promising in silico predictions. Out of the thousands of natural compounds, only a limited number demonstrate good oral bioavailability and t_1/2_ which are crucial for successful oral delivery without any special drug delivery systems. These compounds either remain intact in their original structure or are less metabolized, making them detectable in the blood while exerting a pharmacological effect. Scientists should conduct a systematic literature search before shortlisting natural compounds for further investigation. The critical information to be extracted includes the compounds’ oral bioavailability, half-life (t_1/2_), time to maximum concentration (Tmax), Cmax, area under the curve (AUC), volume of distribution (V_d_), concentration of the compound in tissues to understand how well it is distributed to different parts of the body and any additional relevant parameters if necessary. Concurrently, scientists must compare the pharmacokinetic profiles across different species to gain insights into the potential effects in humans.

## 3. Investigational Compound/Drug Versus Proven Drug(s) for Cancer Treatment

Many published cancer-related drug discovery articles often report investigational drugs alone without proper comparison to established standard chemotherapy drugs. Comparing with standard chemotherapy is necessary to understand how effective the investigational drug is relative to existing treatments. For example, the effect of the decursin compound on prostate cancer cells like PC-3 and DU145 would be more meaningful if it was compared with the standard chemotherapy regimen of docetaxel + prednisolone. Prednisolone is considered the standard of care because docetaxel can cause allergic reactions [24], and prednisolone is not the main chemotherapy agent. Researchers could test decursin + docetaxel + prednisolone against docetaxel + prednisolone alone. However, this is not the best study design because decursin will be metabolized to the active metabolite, decursinol, after oral and intravenous administration. Only decursinol was detected in the plasma of rats. Decursin is a prodrug of decursinol [25]. When taken orally, decursinol is quickly absorbed and has a high oral bioavailability (>45%) [26]. The data from the human study confirmed an extensive conversion of decursin to decursinol, with decursinol having a t_1/2_ around 7.4 h [27]. It is advisable to include decursinol rather than decursin, unless the research focus is on studying the effects of the prodrug. Decursinol is an anticancer investigational compound that has been shown to inhibit prostate subcutaneous tumors and lung metastases grown in mice [28].

Another example involves assessing the combination effect of pyrimethamine and folinic acid on nasopharyngeal carcinoma cell lines or combining pyrimethamine + folinic acid with cisplatin + gemcitabine, compared to cisplatin + gemcitabine alone. Folinic acid (leucovorin) is the standard of care because pyrimethamine can inhibit folic acid and cause toxic effects on the bone marrow [29]. This setup is feasible for both in vitro (where standard of care is optional) and in vivo (where standard of care must be included) studies. In vitro, researchers can utilize freely available tools like SynergyFinder [30] or design studies using clonogenic assays. For in vivo experiments, instead of proposing a full-scale study, a pilot study using mice could be conducted to assess the effects of these combinations before committing to larger studies. This approach offers preliminary findings about efficacy and toxicity while saving time and resources. Before conducting any translational laboratory experiments, it is advisable to consult stakeholders and the pharmaceutical regulatory agency of the respective country. Different countries have varying policies regarding clinical trials, mainly because testing investigational drugs alone in cancer patients may be unethical when proven chemotherapy agents with known efficacy and toxicity profiles are available. For instance, when the author of this manuscript approached the National Pharmaceutical Regulatory Agency (NPRA) to test investigational drug X on prostate cancer patients, NPRA recommended generating more laboratory data based on combining drug X with standard chemotherapy due to ethical concerns. They advised including two research arms: (i) X + docetaxel and (ii) docetaxel, then comparing efficacy and safety in mouse model/s [communicated via an official email from Research and Development Section (rndfarmasi@moh.gov.my) on 2 February 2023@9.59am]. This valuable experience should be shared with the scientific community to raise awareness about testing investigational drugs alone or with/without standard chemotherapy.

**Figure 1:**
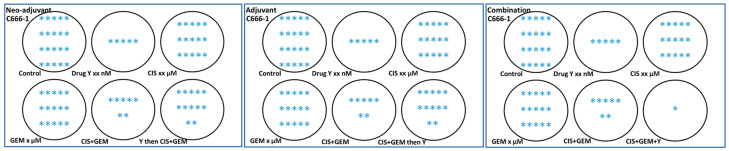
A simple and cost-effective model for testing the suitability of investigational drugs/compounds for neoadjuvant, adjuvant, and combination drug settings. In this model, the C666-1 cells were seeded, and once 80% of cell confluency was achieved, the treatment was started simultaneously for all 3 settings for 6 days. In all settings, 5 wells known as (i) control, (ii) drug Y, (iii) CIS, (iv) GEM, and (v) CIS + GEM are non-modifiable for 6 days. Only the 6th well is modifiable except for the combination setting. In the neoadjuvant setting, the drug Y was first added in the 6th well, and the treatment was carried out for 3 days. Then, the old media was discarded, and new media with CIS + GEM was added and incubated for another 3 days. In an adjuvant setting, the cells were treated with CIS + GEM for 3 days, then the old media was discarded and new media with drug Y was added and incubated for another 3 days. In a combination setting, CIS + GEM + Y was added and incubated for 6 days. For all settings, on day 3 or at 72 h, the media was changed in all wells with respective treatments/controls. The final volume was 2 mL. After the experiment was terminated, the cells were fixed and stained with crystal violet. Based on this representative figure, drug Y is probably not suitable to be given as a neoadjuvant or adjuvant. It is only suitable for combination work. The work requires further preclinical validation which is planned. * Each asterisk represents cancer cells.

On the other hand, an in vitro model is needed to assess whether an investigational drug can be delivered as a neoadjuvant, adjuvant, or in combination with standard chemotherapy, since many clinical trials focus on these settings in patients. The author believes that not all investigational drugs or compounds are suitable for combination with standard chemotherapy. The author studied the potential of drug/compound Y to be delivered as a neoadjuvant, adjuvant, or in combination using an in vitro setup. The experiment was conducted using 6-well plates. The results surprised the author: drug/compound Y is not suitable for the neoadjuvant and adjuvant settings because, unlike the combination setting (where all drugs were added simultaneously), drug/compound Y protects the C666-1 nasopharyngeal carcinoma cells in these contexts, as shown in Figure 1. The manuscript is currently being prepared, and the related results are illustrated in a cartoon format using 6-well plates. While these in vitro findings require validation in preclinical settings, this model could produce preliminary results and enhance our understanding of the drug being studied and whether the investigational drugs or compounds are appropriate for delivery as neoadjuvant, adjuvant, or in combination with standard chemotherapy. Although several in vitro models related to adjuvant or neoadjuvant settings are available [31,32,33,34], based on the author’s search over the past 18 months/1.5 years, no similar design has been found—especially for neoadjuvant and adjuvant settings—similar to the one in Figure 1, where all three settings are designed and experiments are conducted simultaneously. The model designed by the author in Figure 1, which encompasses neo-adjuvant, adjuvant, and combination approaches, should be tested concurrently to obtain preliminary results.

## 4. Route of Drug Administration, Dose Conversion, Frequency of Drug Administration and Study Design for Preclinical Research

The route of drug administration in mice is very important. The method should closely mimic patient experiences in hospitals. It is common in mouse studies that drugs intended for intravenous (i.v.) use are often given via the intraperitoneal (i.p.) route, even though this method is rarely used in clinical settings [35,36,37,38]. The i.v. and i.p. routes are likely different and may lead to inaccurate results. Although administering a drug through i.v. is challenging, it cannot be replaced with i.p. for convenience because this approach impacts the drug’s pharmacokinetics, efficacy, and toxicity profiles. When administered i.p., drugs—especially small molecules often undergo first-pass metabolism, like oral drugs, but the extent of this metabolism via the i.p. route remains unclear. Sometimes, drugs meant for oral delivery are given i.p. to avoid gastrointestinal degradation. Delivering drugs through the i.p. route offers advantages over oral administration, including better absorption and greater stability [35]. Chang et al. [12] found that the maximum tolerated dose of rocaglamide was 4 mg/kg via i.p. and 1.2 mg/kg orally, highlighting that doses vary by administration route. The i.p. route is useful for establishing early pharmacological proof-of-concept [35]. For translational research, the actual route should be used. Ohshima et al. [36], who studied cell-based therapy, noted that i.p. and i.v. routes differ and advised caution when translating animal i.p. results to human i.v. treatments. They also suggested that i.v. experiments in animals are likely necessary before human trials, especially if the clinical route is i.v. In radiotherapy mouse studies, applying the i.p. route instead of i.v. caused excessive radiation absorption in the intestines without affecting tumor targeting, which is relevant for other radiotherapeutic agents [39]. Albendazole, an antiparasitic and potential anticancer drug, shows different outcomes when administered i.p. versus orally in New Zealand white rabbits, with the major metabolite ABZ-SO detected significantly lower via i.p. [40]. While the best route remains debated, researchers should not rely solely on existing literature that may be misleading. Instead, they need to consult their country’s pharmaceutical regulatory agency about the appropriate route- i.p., i.v., or oral-before designing their studies. Understanding the desired efficacy and safety study durations is also critical for successful drug translation.

**Figure 2:**
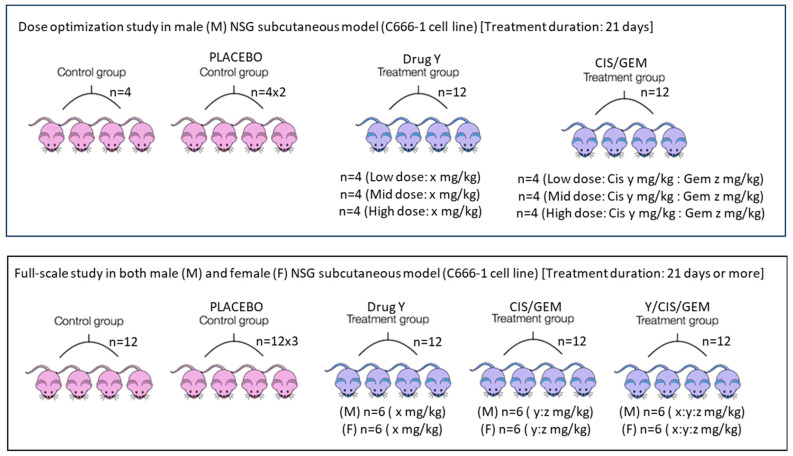
Treatment plans in preclinical experiments. Since nasopharyngeal carcinoma affects more males than females in the ratio of 3:1 [47], the male mice are proposed for the dose optimization study. In case of a full-scale study, both male and female mice must be used. The treatment duration can be adjusted according to how fast the tumor reaches 1500 mm^3^ and the health status of the mice. In case the tumor size is yet to reach 1500 mm^3^, the study must be terminated if the mice already show approximately 20% body weight reduction. The placebo group will receive vehicle control, while the control group will receive neither vehicle control nor the drug. Additionally, a healthy group of mice can be formed, probably with n = 4/gender/group. This setup is important if a blood study needs to be carried out. Alternatively, if permitted by the research committee, include only the standard chemotherapy and the standard chemotherapy plus investigational drug/compound arms, if blood withdrawal is not planned. As previously mentioned, these two groups serve as the primary comparators. **Image credit:** The pink and blue mice images were taken from an online source developed by the members of the teaching team at Harvard Chan Bioinformatics Core and covered by the CC BY 4.0 license for unrestricted use [48].

Apart from the route of drug administration, the dose and frequency used in mice should closely match what cancer patients receive in hospitals. First, the human clinical dose must be converted to an appropriate animal dose, whether oral or i.v. Weng et al. [41] and Manoharan & Ying [6] clearly outline this method. Despite acknowledging differences among species and pharmacokinetics, this approach is chosen to design experiments that are relevant to clinical settings. Designing experiments that harmonize data from both species and pharmacokinetic profiles is very challenging. After the conversion, a dose optimization study is still needed because immunodeficient mice are more fragile. The directly converted human dose can serve as a mid-dose, with one low and one high dose planned accordingly. Dose optimization can be done via pilot study with a small number of mice—probably four per group—over one cycle. If standard chemotherapy involves more than one drug, they can be combined rather than tested separately. The same applies to an investigational drug; if little information is available, its dosage should be determined through a dose optimization study. The goal is to find a dose that effectively shrinks subcutaneous tumors without causing obvious side effects, and mice will remain active after the cycle. During dose optimization, it is acceptable to test doses designed for the investigational drug alone without combining it with standard chemotherapy. Once the optimal dose is determined, a full-scale study can be performed. For nasopharyngeal carcinoma, one chemotherapy cycle lasts 21 days, with cisplatin on day 1 and gemcitabine on days 1 and 8 via the i.v. route [42]. The same setup can be used for mouse experiments in both dose optimization and full-scale studies. Additionally, investigational compound Y, intended for oral administration, can be given daily for 21 days via oral gavage. Based on this information, two main experimental arms can be established: (i) cisplatin (CIS) + gemcitabine (GEM) and (ii) cisplatin + gemcitabine + Y. The two arms mentioned are the primary comparators. The inclusion of control and placebo groups is optional, as in a real-world setting, patients will be recruited within these two main groups. Both genders should be included because nasopharyngeal carcinoma affects males and females equally. It has been reported that a 20 mg/kg i.v. dose of gemcitabine in mice corresponds to a clinical dose; at this level, a Cmax of 67.2 µM can be reached [43]. Clinically, gemcitabine given at 1250 mg/m^2^ results in a Cmax between 50 and 70 µM [43]. Although the typical clinical dose for nasopharyngeal carcinoma is 1000 mg/m^2^, this information guides experimental design. For cisplatin, a dose of 6 mg/kg i.p. can cause kidney toxicity in mice [44]. The clinical dose of 80 mg/m^2^ corresponds to about 26.6 mg/kg in mice when given orally. The estimated injection dose in mice is less than or equal to 8 mg/kg after converting from the oral dose. This conversion depends on oral bioavailability, which is around 31% in rats [45]. It is recommended to use a dose below 6 mg/kg due to the fragility of genetically modified mice used in cancer research, such as NSG, nude, or NOD-SCID mice. These settings are designed to replicate conditions like those experienced by patients in hospitals. Similar setups can be used for safety testing in healthy mice. Figure 2 illustrates the entire process.

**Figure 3:**
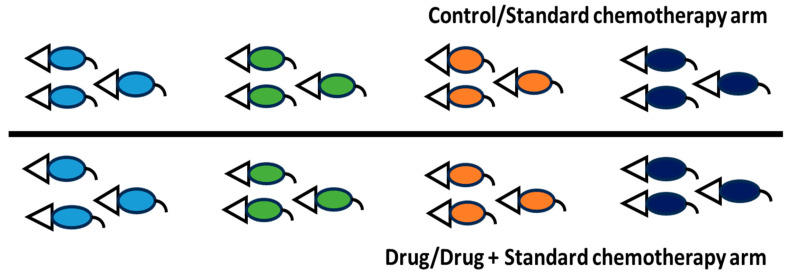
Preclinical mouse study to examine intra- and inter-tumor heterogeneity in cancer research. The light-blue, green, orange, and navy-blue colors represent four different cell lines derived from the same cancer. For example, the colors correspond to HK-1, NPC43, C666-1, and C17 from nasopharyngeal carcinoma, respectively. In this study design, all cell lines are to be subcutaneously inoculated in mice. Once the tumor size reaches 100–200 mm^3^, treatment begins simultaneously. In human clinical trials, results from many patients with diverse backgrounds are typically pooled by research arm and analyzed. The same approach can be used here to mimic a human clinical trial. Use both genders in the study. The ratio can be 1:1 or 3:1 for nasopharyngeal carcinoma, which is mostly diagnosed in males. In this case, the researchers can use n = 4 mice instead of n = 3 in Figure 3, with 3 being male and 1 female (The study can be conducted in both genders, separately to gain more insights). In this study design, the control/standard chemotherapy arm group will only receive vehicle control/cisplatin + gemcitabine while the drug/drug + standard chemotherapy arm will receive investigational compound/investigational compound + cisplatin + gemcitabine. This approach can be used to compare either the “control versus the investigational compound” or “standard chemotherapy versus investigational compound + standard chemotherapy.” The testing will be done on 4 cell lines or more from same cancer. If all four cell lines are not available simultaneously, mouse recruitment can proceed based on their availability. For instance, while waiting for the slow-growing C17 subcutaneous tumors to reach the inclusion size, treatment can commence with the fast-growing HK-1 and NPC43 tumors. During this treatment phase, the C666-1 tumors that meet the inclusion criteria can be recruited and their treatment initiated. It is not essential for all six mice (3 + 3) to be available at the same time. As soon as the inclusion size is achieved for any tumor, it can be recruited, and treatment (either vehicle or drug) can begin. However, a notable disadvantage of this approach is that the overall study duration may be significantly extended, necessitating meticulous documentation to prevent confusion or data mix-ups. This study design (or part of it) was taken/derived/adapted from Crown Bioscience’s “White Paper: The Ultimate Guide to Mouse Clinical Trials” [46].

In another more clinically relevant preclinical study design, particularly for addressing tumor heterogeneity, NSG, nude, or NOD-SCID mice must be injected subcutaneously with different cancer cell lines from the same cancer type, as shown in Figure 3. It is necessary to optimize tumor growth to understand the growth patterns of subcutaneous tumors from various cell lines. For instance, the C17 cell line exhibits a very slow growth rate compared to NPC43 and C666-1 after inoculation with 1 × 10^6^ cells. Based on the author’s experience, it takes less than 2 weeks to reach 30 mm^3^ for NPC43 and C666-1, with consistent growth in nearly all mice, whereas it takes about 40 days to reach 20 mm^3^ for C17, which shows inconsistent growth in mice after inoculation with 1 × 10^6^ cells combined with Matrigel (1:1) with at least 95% cell viability. If a tumor grows slowly, cell inoculation must be performed much earlier, before the development of fast-growing subcutaneous tumors. Once tumors reach approximately 100–200 mm^3^ or 80–150 mm^3^, mice should be randomized using related scientific software and assigned to different study groups. The treatment then proceeds for one or two cycles. At the end of the study, tumor volume in mm^3^ and body weights can be collected and analyzed together. Alternatively, mice can be recruited according to the study group as tumors develop and reach the inclusion size of roughly 80–100 mm^3^. This method more closely mimics actual clinical trial conditions. The disadvantage of the first approach is that researchers may find it difficult to produce uniform subcutaneous tumor sizes across at least three or four cancer cell lines due to differing growth rates. While the second approach can address this issue, it presents challenges related to documentation. The study design was taken/derived/adapted from Crown Bioscience’s “White Paper: The Ultimate Guide to Mouse Clinical Trials” [46].

## 5. The Age, Gender, Strain of Mice, and the Importance of the ARRIVE Guideline in Preclinical Studies

**Figure 4:**
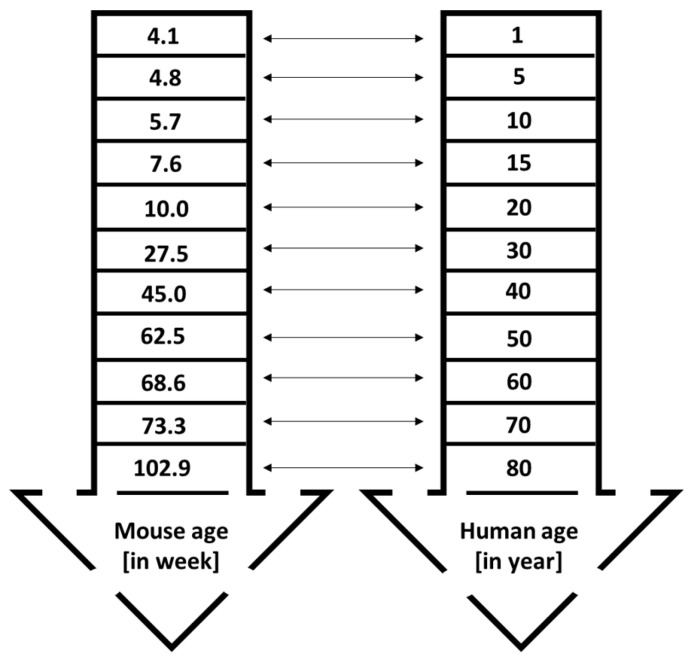
The comparative age equivalence between mice (in weeks) and humans (in years). The figure was generated from data available in Wang et al. [52]. The design of this image was adapted from Manoharan [49] with some modifications to avoid image similarity.

Cancer is still regarded as an old-age disease that affects both males and females. It is usually diagnosed at age 50 and older, although the incidence trend among younger populations is increasing [49]. For example, in the United States, 90% of cancers are detected in individuals aged 50 or above. Only 1.7% of cancer-related deaths in both genders are reported in populations below 40 years old. The median age of cancer diagnosis is 66, with breast, colon, lung, and prostate cancers having median ages of diagnosis at 62, 67, 71, and 66 years, respectively [49,50]. This data is crucial for designing animal studies, as the physiological functions of young and old mice differ. Preclinical studies often overlook the age and gender of mice. Both genders should be included, except for gender-specific cancers like prostate and ovarian tumors. Results from one gender cannot be applied to the other. For instance, efficacy and safety data from female mice cannot be assumed to mirror effects in male mice. In 2014, the National Institutes of Health (NIH) announced the requirement to include both sexes in preclinical research [51]. One reason researchers often prefer female mice over males in cancer studies is cost—male mice tend to fight, making maintenance more difficult and expensive [51]; however, research involving both sexes remains essential. In preclinical studies, young mice between 4 and 8 weeks old are commonly used, as reported in a systematic review. Of 14 studies, only three used male mice [6]. According to Wang et al. [52], mice aged between 4 and 8 weeks are equivalent to approximately 1–15 human years, which is juvenile age (Figure 4). This age range is suitable for studies on juvenile cancers, such as childhood leukemia. The researchers noted that using mice from this age group might hinder future clinical trial success. For efficacy and toxicity assessments, it is recommended to use mice at least 28 to 60 weeks old, roughly corresponding to 30 and 50 human years, respectively. Mice younger than 25 weeks and older than 25 weeks have different physiological characteristics. Young mice with fully functioning organs may appear to have lower toxicity risks, but older mice could display increased vulnerability [49], complicating the translation of research to humans. Yanai & Endo [53] reported that mice’s bodily functions decline progressively starting at 24 weeks (6 months). This finding supports the use of mice older than 25 weeks for improved relevance in research. Maintaining mice for 60 weeks is costly but necessary for rigorous science and translational research. If keeping mice that long is not feasible due to staffing, operating costs, or facility limitations, older mice can be purchased from reliable suppliers. For example, The Jackson Laboratory [54] provides both male and female C57BL/6J mice aged between 25 and 90 weeks. Using these mice allows for toxicity studies on geriatric animals at least 60 weeks old.

It is important to note that C57BL/6 mice are not the only strain used in cancer research. The C57BL/6 mice provide many benefits for doing research in the fields of physiology, immunology, safety and efficacy, cancer, genetics, and mouse model development. In addition, this breed of mice is often used for syngeneic tumor research. Even though C57BL/6 mice have a functional immune system and would reject xenografts originating from humans, they offer useful information regarding the interaction between the immune system and tumors [55]. Regrettably, in certain countries, the results of cancer therapy using syngeneic tumors may not be considered valid for translating preclinical research into human clinical trials, even though the development of tumors in mice and humans has shown comparable histological characteristics [52]. The reason is that, although the human and mouse genomes are 99% similar, the tumors developed in syngeneic C57BL/6 mice use mouse cancer cells instead of patient-derived xenografts or cell lines. The syngeneic tumors are less likely to be rejected due to their origin from the same species. This distinction raises the issue of whether it is suitable to use syngeneic mouse cancer models when human cancer cell lines are readily available and can be successfully grown in immunodeficient mouse strains, which are different from immunocompetent C57BL/6 mice. Commonly used strains of immunodeficient mice in cancer research include NOD-SCID-Gamma (NSG) mice, NOD-SCID mice, nude mice, and a few more strains. These mouse strains exhibit lower to moderate rates of rejection of human cancer cells compared to C57BL/6 mice. They can be effectively used to develop human tumors and can be treated with cancer therapies. One significant drawback of using immunodeficient mice is that they present challenges in studying cancer immunology, primarily due to the limited availability of immune cells in these strains. Another significant drawback of using immunodeficient mice is their heightened susceptibility to infection if the surrounding habitats are not sufficiently clean. In NSG mice, there is a lack of significant immune cells, but in nude mice, there are functioning B cells and robust natural killer (NK) cells [56]. These differences not only dictate the susceptibility of different strains to infection but also influence the rejection rate of human cancer cells in various mouse strains. It is predicted that nude mice would have somewhat higher rejection rates compared to NSG mice. According to the author’s preclinical experience, establishing stable tumors using the NPC43 nasopharyngeal carcinoma cell line in nude mice presents significant challenges. This difficulty arises primarily from the inconsistency in tumor size and the time taken to achieve 100 mm^3^ (approximately 60 days) when compared to the use of NSG mice (<20 days). Additionally, the therapy showed a more favorable response in nude mice with nasopharyngeal tumors compared to NSG mice, perhaps because of the presence of mature B cells and NK cells. The administration of drug Z in NSG mice could not decrease the tumor volume/size of a recently developed patient-derived xenograft (PDX) from a Malaysian nasopharyngeal carcinoma patient, which was grown in mice. However, drug Z effectively suppressed it in nude mice. In addition, nude mice exhibit greater tolerance to larger doses of therapy in comparison to NSG mice. When administered at a dose of 45 mg/kg, drug Z caused a substantial decrease in body weight in NSG mice compared to nude mice. Simultaneously, it is important to consider the age of nude mice, as they may generate a certain number of populations of extrathymic T cells (“leaky”) that can reject tumor grafts, especially in older mice [57]. The average lifespan of nude mice typically ranges from 6 months to 1 year. Nevertheless, nude mice can endure an extended period, resembling the lifespan of regular mice (18–24 months), when they are exposed to highly sanitized and germ-free surroundings and receive antibiotics [58]. While nude mice may seem appealing for cancer research, they are not suitable for long-term studies. Other strains for consideration are the SCID mice which have normal NK cells and macrophage activity with T and B cells leakage in age-increasing mice, the NOD-SCID mice which have reduced NK cells and macrophage activity with T and B cells leakage in older mice and have a life span of 8 months. The NOD/SCID rg^null^ mice which can be divided into two groups known as NOG (bind to cytokines but unable to transduce the signal) and the NSG (the most immunodeficient mouse strain) are known to be the best models to transplant human cancer cells and tissues but lack immune cells which are needed in therapeutic studies involving immunomodulatory drugs [59]. To carry out a comprehensive assessment of long-term efficacy and toxicity studies, researchers must carefully evaluate the benefits and drawbacks associated with each mouse strain. This information helps researchers select an appropriate mouse strain for initiating cancer preclinical studies. In case a tumor does not grow in one strain, it is advisable to repeat the experiment in at least another mouse strain. This information applies to the efficacy study as well. Indeed, mouse strains play a role in human tumor development and efficacy studies.

Preclinical studies serve as the crucial link between in vitro and clinical studies, playing a key role in research translation. Therefore, preclinical research should follow guidelines to produce transparent results. In clinical trials, adherence to guidelines, along with randomization and double blinding (or at least single blinding) with appropriate sample sizes, often influences policy. Due to their low risk of bias, such studies typically enjoy high trust. Begley and Ellis [60], in a comment published in Nature, pointed out that a major reason for failures in cancer-related clinical trials is the quality of preclinical data. Drug development depends heavily on published literature. Because clinical trials are complex, solid preclinical studies are essential. This manuscript further elaborates on some points discussed by the authors. One way to improve preclinical studies is by adopting the Animal Research: Reporting of In Vivo Experiments (ARRIVE) guideline, developed in 2010 to help researchers improve the reporting quality of preclinical experiments [61]. An article in PLOS Biology states that randomization, blinding, and sample size calculations are reported in only about 30%, 20%, and 10% of preclinical publications, respectively [61]. These parameters are all critical in preclinical studies. The main challenge in fully following the ARRIVE guideline is manpower, especially for small research teams. For a small team, using online software for mouse randomization is feasible. Regarding blinding, the drug administrator should not measure tumor size or mouse weights to avoid bias. A team of two to three researchers should at least meet these two criteria. For larger teams (4–6 members), tumor harvesting should be blinded by having a different person perform the procedure, and data analysis should be independent. If possible, an independent researcher should handle drug preparation instead of the drug administrator. The ARRIVE guideline contains 20 items, with 10 essential and 10 recommended for transparent reporting in preclinical studies. It helps researchers design their experiments and guides writing to ensure transparency. Following the ARRIVE guideline can improve study design and increase trust in the research outputs by reducing bias. However, not all preclinical researchers are aware of the guideline. The author hopes this article raises awareness of the ARRIVE guideline. The author believes that adhering to it will improve both the quality of preclinical research and the success rate of translational research.

## 6. Subcutaneous Versus Metastatic Mouse Models in Preclinical Studies

Many cancers are highly metastatic, meaning they can spread from the original or primary site to secondary sites, such as other organs, making the disease and its treatments more complicated. Metastasis causes 90% of cancer-related deaths [62]. Most published articles on drug discovery use only subcutaneous tumor models. Orthotopic models are less common due to the complex anatomical locations of certain cancers and require highly skilled staff. For example, unlike injecting breast cancer cells directly into the mouse mammary gland, injecting nasopharyngeal carcinoma cells into the mouse’s nasopharynx is extremely challenging. This difficulty makes the subcutaneous model more attractive for cancer studies. The subcutaneous tumor model is an ectopic model where cancer is grown at a site different from the original [63]. The environment of subcutaneous tumors is likely quite different from the original site because of the nature of the location. For example, subcutaneous tumors derived from PC-3, MCF-7, and C666-1 cell lines, representing prostate, breast, and nasopharyngeal carcinomas, respectively, are unlikely to reflect the original environments. Subcutaneous tumors are isolated and encapsulated, and they do not exactly represent human disease [64]. They are also less likely to metastasize compared to orthotopic models [65]. The popularity of subcutaneous tumor models is mainly due to their high visibility to the naked eye, simple development protocol with a high success rate, straightforward measurement of tumor size, and reproducibility [65,66]. Additionally, mice typically do not experience major stress, such as from surgery or pain. This technique can also help prevent the loss of animals. Despite these advantages, relying solely on the subcutaneous model provides a less comprehensive understanding, although metastatic prostate (such as PC-3 and DU-145) and nasopharyngeal carcinoma (C17) cell lines are often used to develop subcutaneous tumors. At least one metastatic model is necessary. One common metastatic model is the lung metastasis model, as the lungs are among the most frequent sites for metastasis [63]. To develop metastasis models, researchers often use imaging systems to track the development of metastasis in mice after injecting cancer cells labeled with green fluorescent protein (GFP) or luciferase-luciferin, which are widely used and well-established [67]. Although this method is advanced, it is expensive due to the need for specialized imaging systems and related scheduled maintenance, additional equipment for cell transfection and sorting, and highly trained personnel. Not all countries have access to such advanced equipment, but cancer metastasis remains a universal disease. Instead of relying on expensive equipment, traditional methods can be used to develop cancer metastasis models. For example, Zeng et al. [68] reported developing a lung metastasis model for nasopharyngeal carcinoma by injecting the C666-1 cell line into the lateral tail vein of mice. This injection often leads to lung metastasis, depending on the number of cells injected. For example, Smith et al. [64] could not detect lung metastasis after inoculating with 2 × 10^5^ C666-1 cells/NSG mouse into the tail vein and incubating for 12 weeks, while Zeng et al. [68] detected lung metastasis nodules at 1 × 10^6^ cell injection after incubating for 30 days. If it was not previously described for the cell line of interest, researchers can design a lung metastasis model using mouse groups (n = 6 per group). Every week (or every two weeks for slow-growing cancer cell lines), one group of mice will be sacrificed, and their lungs will be harvested, processed, and stained with H&E. For more comprehensive detection, it is preferable to prepare five slides from different sites on the same whole lung. Alternatively, the author of this manuscript cut the whole lung into 5 lobes—right lung (superior lobe, middle lobe, inferior lobe, post-caval lobe) and left lung—left lobe [69] and each lobe has 5 slides which is equal to 25 slides/whole lung for in-depth detection of metastasis especially in detecting micro-metastasis/nest of cancer cells. A histopathologist will review the slides for any signs of metastasis. Metastasis development can be categorized into two types: micro-metastasis and macro-metastasis. It is recommended to develop a micro metastasis model if the drug discovery process spans 45 to 90 days. The macro-metastasis model generally does not support long-term studies, though the duration depends on the mouse strain used except if mortality-related study design (and for analysis) is included. In a mortality-related study design, it is theoretically expected that control mice will die earlier than those in the treatment group before reaching the endpoint, assuming the treatment is effective.

Although this method seems attractive, it is possible for the mice to die immediately or soon after tail vein injection. Despite the successful establishment of the C666-1 lung metastasis model by Zeng et al. [68] in 6–8 weeks old female nude mice, the author of this manuscript was unable to replicate similar success in 56-week-old NSG male and female mice after inoculating them with 1 × 10^6^ C666-1 cells [passage number 63 or P63; cell viability was 94% in phosphate-buffered saline (PBS) after filtered using 40 µm cell strainer; final volume of cell suspension was 200 µL/mouse; needle size was 26 G; time taken from syringe preparation to start inoculation was <2 min; Animal Care and Use Committee (ACUC) approval number: ACUC/KKM/02(03/2025); Institutional Biosafety Committee (IBC) approval number to use NSG mice as living modified organism (LMO): JBK (S) 600-3/1/206 (8)] and injecting them very slowly into the tail vein over 30 s (similarly tested over 60 s). Severe weakness (in most of the cases) and sudden death (in a few cases) occurred with 0% success rate even under the microscope the 200 µL cell suspension did not contain clumps before tail vein injection. Only one mouse was active for 20 min, after which it exhibited mild followed by severe weakness. Necropsy revealed that the right ventricles of the hearts of all mice were enlarged, potentially resulting from cell clotting or clumping. At a concentration of 2.5 × 10^5^ C666-1 cells per NSG mouse, the author found that 75% of the mice remained active following intravenous tail vein inoculation with one mouse exhibiting weakness, managing to move a few steps before pausing and repeating the process, accompanied by heavy breathing. The author’s current work especially the usage of 2.5 × 10^5^ C666-1 cell concentration/NSG mouse aligns with Smith et al. [64], who reported the successful inoculation of 2 × 10^5^ C666-1 cells per NSG mouse. It is likely that the mouse strain and related cell concentrations significantly influenced the observed outcomes. The author has reached out to the corresponding authors of Zeng et al. [68] via email (on 1 December 2025) to request advice and further guidance on developing the C666-1 lung metastasis; however, as of 17 December 2025, no response has been received. The author has previously established lung micro metastases using NPC cells in NSG mice [(ACUC approval number: ACUC/KKM/02(06/2023); IBC approval number: JBK (S) 600-3/1/128 (7)], in which 1 × 10^6^ of the EBV^+^ P53^mutant^ NPC cells were administered into the tail vein over a duration of 30 s. Note: The author is unable to share the name of the cell line as it is intended for in-house experiments. The author is willing to share the H&E images of the nest of cancer cells in the lungs, provided that a reasonable request is made and approved by both the Director General of Health Malaysia and the Director of the Institute for Medical Research. Based on these experiences, it is advisable to design a pilot study to assess whether the cell line or PDX cells are suitable for delivery into the tail vein to induce lung metastasis. For liver metastasis development using a non-imaging method, surgery must be performed to expose the liver and inoculate it with cancer cells. This approach is also known as the orthotopic method for creating liver-origin cancer models. The outlined steps should be followed. Injecting cells into the spleen can result in the development of liver metastasis. However, this procedure is technically demanding, as it necessitates general surgery to expose the spleen and requires the removal of the spleen one minute after the cell injection. Furthermore, adequate care must be provided to the mice [70,71]. The advantage of this traditional lung metastasis model is that it does not require advanced, costly imaging systems and uses parent cancer cells without modification, such as GFP tagging which may require approval from the IBC for use. Simple H&E-stained slides are enough for metastasis detection by a histopathologist. The main disadvantage is that more mice are needed during the optimization stages. Once optimized, the number of mice can be reduced, and the main experimental plan can be established. Results from both subcutaneous and metastasis models will further strengthen the pathway toward successful research translation.

Another important aspect that researchers often neglect is the use of authentic cell lines in cancer studies. Cell line authenticity is crucial for ensuring legitimacy and scientific reproducibility. Validated cancer cell lines maintain the traits of their original tumors and serve as vital resources for research. Experimental results are verified across multiple cell lines to ensure that the effect observed in a single cell line is not representative of the disease as a whole [72]. Over 500 cell lines are listed in the misidentified cell line registry maintained by the International Cell Line Authentication Committee (ICLAC), and more than 900 cell lines are classified as problematic in the Cellosaurus database [72]. For example, 5-8F, 6-10B, CNE-1, CNE-2, HNE-1, HONE-1, SUNE1 and SUNE2 are the so-called cell lines for nasopharyngeal carcinoma research that are contaminated with HeLa and include extra genetic material that originates from a source that has not been recognized [73]. So far, many research groups studying NPC have continued using these cell lines despite genetic testing revealing they are contaminated with HeLa cells. From 2000 to 2023, 1282 papers reported using NPC cell lines contaminated with HeLa cells. If we assume that 10–12% of all NPC cell lines used worldwide are cross-contaminated, it indicates that hundreds of millions of dollars may have been wasted in the biomedical and healthcare industries globally due to inaccurate or misleading information [73].

## 7. Comorbidities and Related Drug Studies in Preclinical Models

A comorbidity in cancer-diagnosed patients is commonly defined as the co-occurrence of a disorder or chronic disease alongside cancer. Common chronic conditions include high blood pressure, diabetes, and organ-related diseases such as those of the heart, kidneys, and liver, as well as mental health disorders, among others. It has been projected that at least 75% of cancer patients have one or more comorbidities [74]. The most common comorbidities among cancer patients in the United States, China, India, and Nigeria are high blood pressure and diabetes. Age positively correlates with these conditions, increasing the likelihood of comorbidities in older cancer patients [75]. Experiencing one or more comorbidities can affect the patient’s overall cancer treatment outcomes, often resulting in poorer survival rates and lower quality of life [76,77]. Despite the common occurrence of one or more comorbidities in cancer patients, this situation is often overlooked or not well understood in preclinical drug discovery experiments, especially in mouse models. This experiment is important because the interaction of high blood pressure and diabetes drugs, such as amlodipine and metformin, with standard chemotherapy for nasopharyngeal carcinoma—namely cisplatin combined with gemcitabine and investigational drugs like pyrimethamine may lead to different outcomes regarding tumor size, shrinkage, overall survival, and toxicity in mouse models compared to testing without these medications. Drug–drug interactions pose a significant risk to roughly 30% of cancer patients who are on multiple prescriptions. Combining different drugs can alter their pharmacokinetic profiles. Some drugs can impair the absorption of others, affecting their volume of distribution and drug-protein binding when they interact. Additionally, drugs can disrupt the activity of cytochrome P450 (CYP) enzymes in the liver, potentially increasing or decreasing the drugs’ effects and related toxicities [78]. It has been reported that the investigational anticancer compound resveratrol affects the pharmacokinetics of erlotinib, a tyrosine kinase inhibitor, in rats. Erlotinib has a narrow therapeutic window, and this alteration could influence the drug’s efficacy and toxicity when administered with resveratrol [79]. In another study, potential drug–drug interactions were observed in pediatric hemato-oncology patients. About 68% of these patients experienced at least one significant interaction, often related to comorbidities (*p* = 0.019) [80]. Testing common drugs for comorbidities, such as amlodipine and metformin, together with cancer-related drugs (investigational drugs with or without standard chemotherapy), is an important step. The testing can be divided into two parts: efficacy and safety. These studies do not need to be the main focus of the research project. Instead, they can be designed as sub-studies that provide additional data for better understanding. For example, in an efficacy study, a subcutaneous tumor model can be used. If the authors used four cell lines in the main in vivo project, one cell line can be selected for testing an investigational drug (with or without standard chemotherapy) plus drugs for comorbidities. It is possible to create a diabetic model in NSG mice since NSG mice are sensitive to streptozotocin (STZ) [81], but this process is tedious. Undeniably, a diabetic cancer mouse model is more advanced because diabetes itself can promote cancer growth [82]. To simplify methodologies, only drugs are administered to NSG or nude mice bearing subcutaneous tumors, and effects are observed for at least two cycles (21 days per cycle for nasopharyngeal carcinoma), until the tumor reaches approximately 1500 mm^3^, or until toxicity-related side effects, such as a 20% reduction in body weight, develop whichever occurs first. Next, healthy mice without tumors are typically used for comprehensive safety studies, including blood tests, tissue analyses, and other assessments required for first-in-human testing. This is not only a requirement but also necessary because human tumors cannot be generated in non-immunodeficient mice due to rejection by intact immune cells. Immunodeficient mice like NSG (living modified organism-LMO) are considered weak mice, and toxicity results from NSG mice can serve as supplementary data to make a preliminary decision before proceeding to a full-scale safety study in healthy mice such as CD-1 or C57BL/6 mice, which are usually used for toxicity testing. The same steps as in the efficacy study should be repeated in safety studies over 90 days, preferably (or obligatorily in certain countries) conducted in a good laboratory practice (GLP)-certified laboratory. Mice are housed individually in cages. After completing the studies, tests such as full blood count, liver function, renal profiles, cardiotoxicity-related assessments, immune-related toxicity tests, and other tissue analyses should be performed. Compiling these comprehensive data will help determine the suitability of the drug(s) for translational research.

## 8. The Acidic Tumor Microenvironment (TME) and the Efficacy of the Drug Candidate

Acidic TME is a hallmark in solid cancers, contributing to cancer progression. The acidity of TME results from the high glycolysis activity of cancer cells, even in the presence of sufficient oxygen in the tumor. During glycolysis, glucose is converted to lactate, which is then secreted into the extracellular TME. Lactate accumulation influences TME acidity. Additionally, the lack of a functional lymphatic drainage system significantly contributes to extracellular acidity in TME. The pH of extracellular TME typically ranges between pH 6.5 and pH 6.8 [83], with a report noting it can be as low as pH 5.5 [84]. Elevated lactate levels in the extracellular TME activate the GPR81 receptor, which further increases glycolytic activity and promotes acidification. The acidic TME not only accelerates tumor progression but also suppresses key immune cells such as T- and NK-cells while attracting immunosuppressive cells [85]. Furthermore, acidic extracellular pH promotes p-glycoprotein expression, enhancing drug efflux by tumor cells and leading to drug resistance [86]. The acidic TME can alter the structure and charge of anti-cancer drugs. These changes can hinder the drug’s uptake by tumor cells and reduce their effectiveness [87]. For example, doxorubicin becomes highly charged, which causes problems with its absorption by cancer cells in the acidic TME [88]. Basic anti-cancer drugs are mostly affected by the acidic TME, while acidic and neutral drugs are less impacted. Drugs like anthracyclines, anthraquinones, and vinca alkaloids being basic are influenced by the acidic TME, which prevents them from reaching cancer cells and diminishes their cytotoxic effects [89]. Additionally, the acidic TME can negatively impact gemcitabine, another weak basic drug [90]. Gemcitabine is an important chemotherapy agent used to treat many cancers, including pancreatic and nasopharyngeal carcinomas. Acidic TME too can cause radiotherapy resistance. Oxygen must be present during radiation therapy for it to produce free radicals that damage DNA and cause cell death. There might not be enough oxygen for this radio-sensitization in hypoxic environments [88]. Thyroid adenocarcinoma and chronic lymphocytic leukemia had the lowest hypoxia ratings, whereas lung and cervical squamous cell carcinoma are considered the most hypoxic cancer types [91]. The hypoxia ratings were determined using 15 top-ranked hypoxia-related genes, namely, *VEGFA*, *SLC2A1*, *PGAM1*, *ENO1*, *LDHA*, *TP11*, *P4HA1*, *MRPS1*, *CDKN3*, *ADM*, *NDRG1*, *TUBB6*, *ALDOA*, *MIF*, and *ACOT7* [91]. The collective of these genes is also known as Buffa signature [91]. On the other hand, the acidic extracellular pH can result in the release of (cysteine or aspartyl) cathepsin proteinase activity in vitro, which has been suggested to play a role in local invasion and tissue remodeling. Additionally, cells subjected to low pH conditions in vitro demonstrate greater invasion, both in vitro and in vivo [92]. Since cancer cells have a greater ability for evolution, they are able to establish adaptation mechanisms that enable them to survive as well as multiply in surroundings that are acidic [92]. These are the several important reasons behind the need to test anticancer investigational drugs or compounds (with or without standard chemotherapy) in in vitro acidic tumor microenvironments.

**Figure 5:**
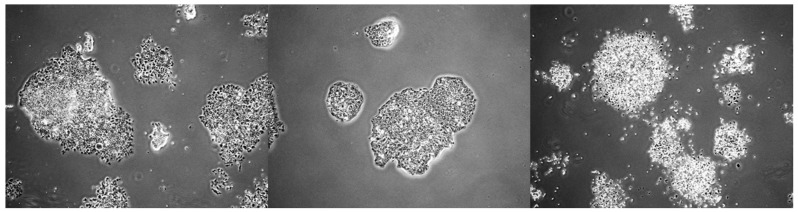
The morphology of three nasopharyngeal carcinoma-related clones—NPC43, C17, and C666-1—was observed on day 21, following clone culture period of three days in media with pH 6.5 and 5% serum, prior to the commencement of treatment. Unlike 2D monolayers, most of the visible clones have multiple layers of cells. The images were taken using a 4× microscope lens.

It is extremely difficult or impossible to fully model TME in vitro. Most or nearly all in vitro studies use culture media at pH 7.4. At this pH, the drug’s actual effect on cancer cells often remains poorly understood and may be misleading, especially for basic drugs. This initial in vitro step could lead to a failure in translational research. Although accurately modeling the complete TME on a cell culture plate is very challenging, two main TME conditions can be simulated to test drug efficacy: adjusting the pH of the media and serum concentration to more acidic levels (pH 6.5) and at least 50% of serum reduction, respectively. Hypoxia can be created using a hypoxia chamber. Some optimization may be necessary because different cancer cells have unique characteristics. The two-dimensional (2D) setup is probably not ideal. A three-dimensional (3D) model, such as spheroids, provides a more useful starting point. Additionally, cancer clones developed over three weeks or more can serve as a viable platform for studying anticancer drug efficacy in a TME-mimicking condition. These clones can be generated via a clonogenic assay, where a small number of cells are seeded, and after forming large visible colonies, they are used for efficacy testing (Figure 5). For instance, 8000 NPC43 cells (from nasopharyngeal carcinoma) can be seeded in a 6-well plate, with regular media changes at pH 7.4 for 14 days. On day 14, the pH and serum concentration are gradually reduced over a week to reach a final pH of 6.5 and 5% serum. The clones are incubated at pH 7.1 for two days, then at pH 6.8 for another two days, and finally at pH 6.5 for three days before starting treatment. Serum concentrations are also gradually lowered together with pH. Sudden pH changes should be avoided to prevent shock, as acidity in the tumor microenvironment naturally develops over time. On day 21, treatment begins and lasts for 3–5 days without media changes. Afterward, the stained clones can be destained, and their absorbance measured. Estimating clone numbers may be inaccurate because treatment effects can cause large clones to break apart into smaller ones. Big clones with multiple layers of cells at day 21 are essential, as late-stage (stage 3 and 4) disease is common. Results are compared against conditions of pH 7.4 and normal serum levels. These setups can support various in vitro assays, which reveal drug efficacy based on these experimental conditions.

## 9. Discussion and Conclusions

Study designs for research activities are primarily influenced by published articles. Before selecting articles to serve as references for guiding these research activities, researchers must assess the quality of the published work. Errors at this stage can significantly delay the research process or result in inaccurate outcomes and related conclusions. For instance, in addition to the issues discussed in Section 2, Chang et al. [12] reported that a single oral dose of 5 mg/kg (administered in 20% DMSO in saline) resulted in a therapeutic dose of 20–50 nM in NSG; however, the maximum tolerated dose (MTD) was only 1.2 mg/kg (in 30% hydroxypropyl-β-cyclodextrin (HPβCD) which was used for subsequent experiments. While this study design provides some information such as 50% oral bioavailability and suitability to dose rocaglamide once daily, it raises questions, particularly regarding the authors’ choice to use 30% HPβCD instead of 20% DMSO in saline. The methods employed are not consistent across both oral concentrations. Loftsson et al. [93] mentioned “in the case of oral administration, volume for dilution/dissolution of the complexes is relatively low and hence excess CD can hamper drug absorption from the gastrointestinal (GI) tract”. It is not known whether rocaglamide was fully released from the drug-CD complexes. This raises further questions about whether 1.2 mg/kg is indeed the MTD.

Simultaneously, the researchers should evaluate the quality of systematic reviews and meta-analyses before including them to help identify research gaps. Careful evaluations are essential to ascertain the appropriateness of a meta-analysis, rather than proceeding with it by default. This involves assessing whether the studies are adequately comparable in terms of their design and implementation (apple compared with apple; orange compared with orange), whether there exists a significant risk of bias, and whether any statistical heterogeneity has been adequately addressed [94,95]. The authors should also assess whether the articles involved pooling randomized controlled trials with non-randomized controlled trials. Combining these two types of trials should be avoided, as they have different risk profiles for biases, and the robustness of their methodologies differs significantly [96]. In PLOS Computational Biology, it was mentioned that although meta-analyses are intended to occupy the pinnacle of the evidence hierarchy, they often fall short of the expected standards and do not consistently exhibit rigorous methodological quality [97].

Basic to translational studies (bench to bedside) mainly involve huge investments. The results from advanced basic cancer research methods, such as various types of 3D cell culture, the use of valuable organoids, organs, or tumors on chips, treatment-related omics research, advanced imaging techniques, cutting-edge preclinical models and others, could be inaccurate or meaningless if the fundamental study design, especially involving drugs or compounds, is not properly planned. In this manuscript, the author discusses several important points that the author believes can positively impact laboratory-based study designs and related outputs, with the hope of improving the success rate of translational research.

## 10. Future Directions

Translational research requires a fresh perspective because it is becoming more costly. Less than a dollar of value is returned for every dollar spent on research and development [2]. There are significant gaps in current laboratory-based cancer drug discovery-related study designs that could be addressed to enhance the success rate of translational research. Research efforts should maintain a high standard and continue to build a legacy, rather than coming to an abrupt halt upon project completion, unless unforeseen circumstances arise, such as high toxicity and/or low drug potency. It is essential to publish negative results to prevent redundancy, even though it can be challenging to secure publication of such findings in prominent journals. At the very least, negative results should be disseminated as preprints to inform the research community. Furthermore, research grant committees, funders, stakeholders, journal editors, and reviewers should be aware of the need to refine the criteria for producing and approving well-designed, high-quality research proposals and manuscripts, which could ultimately enhance the success rate of translational research.

## Data Availability

The raw data supporting the conclusions of this article will be made available by the authors on request.
